# Left atrial volume as an independent predictor of exercise capacity in patients with isolated diastolic dysfunction presented with exertional dyspnea

**DOI:** 10.1186/1476-7120-12-19

**Published:** 2014-06-14

**Authors:** Nithima Ratanasit, Khemajira Karaketklang, Srisakul Chirakarnjanakorn, Rungroj Krittayaphong, Decho Jakrapanichakul

**Affiliations:** 1 Division of Cardiology, Department of Medicine, Siriraj Hospital, Mahidol University, Bangkok 10700, Thailand; 2 Department of Medicine, Siriraj Hospital, Mahidol University, Bangkok 10700, Thailand

**Keywords:** Left atrial volume, Exercise capacity, Diastolic dysfunction, Dyspnea

## Abstract

**Background:**

Left atrial volume (LAV) and exercise capacity are important prognostic determinants of cardiovascular risk. Exercise intolerance and increased LAV are expected in patients with diastolic dysfunction. While dyspnea is the symptom reported by the patient and considered subjective, exercise capacity obtained by exercise testing provides an objective measure of cardiovascular fitness. The objective of this study is to determine the relationship between LAV index and exercise capacity in patients with isolated diastolic dysfunction who presented with exertional dyspnea.

**Methods:**

We studied consecutive patients with dyspnea who underwent treadmill exercise testing and transthoracic echocardiography on the same day. LAV was assessed using the biplane area-length method. Symptom-limited exercise testing was performed immediately after echocardiography. Patients with coronary artery disease, valvular or congenital heart disease, left ventricular systolic dysfunction, pulmonary hypertension or positive exercise test were excluded.

**Results:**

The study consisted of 111 patients (58.1 ± 9.2 years of age, 54.1% male, 64% hypertension, 57.7% dyslipidemia and 20.7% diabetes). The exercise duration and capacity were 6.8 ± 2.1 minutes and 7.7 ± 1.9 METs, respectively. Left ventricular ejection fraction and LAV index was 71.0 ± 5.8% and 31.4 ± 10.5 ml/m^2^, respectively. In multivariate analysis, age [odds ratios (OR) 0.94; 95% confidence interval (CI) 0.89-0.99], body mass index (OR 0.82, 95% CI 0.72-0.93), and LAV index (OR 0.92, 95% CI 0.87-0.97) were associated with good exercise capacity.

**Conclusion:**

In patients with isolated diastolic dysfunction and exertional dyspnea, an increased LAV index, a marker of chronic diastolic dysfunction, is associated with poor exercise capacity.

## Background

Exercise capacity has been known to be an important prognostic determinant of cardiovascular and overall mortality [1,2]. The peak exercise capacity achieved during exercise testing is even more powerful than other established risk factors for cardiovascular disease such as hypertension, smoking, and diabetes mellitus [1]. Dyspnea is a classic symptom of heart failure. However, it is a subjective symptom with multiple potential etiologies, not solely due to increased filling pressure. It may be a manifestation of diastolic dysfunction or other non-cardiac conditions, such as pulmonary disease, obesity, anemia, endocrine disorders, physical deconditioning, or psychological disorder, and may as well limit exercise tolerance. Several studies have provided data on the significance of left ventricular (LV) diastolic dysfunction. The number of patients with heart failure in the presence of preserved systolic function was reported in 30-40% and LV diastolic dysfunction may be the underlying cause of symptoms in such patients [3]. While dyspnea is the subjective symptom reported by the patient, exercise capacity obtained by exercise testing provides an objective measure of quality of life, physical-activity status and cardiovascular fitness. Poor physical fitness is a modifiable risk factor, and the improvement in cardiovascular fitness has been demonstrated to improve prognosis [4].

Previous studies have shown that LV diastolic function is an important determinant of exercise capacity in normal individuals and patients with various heart diseases [5-10]. The assessment of LV diastolic function can be performed noninvasively by the conventional echocardiography and tissue Doppler imaging (TDI). Left atrial volume (LAV) has been recognized as an indicator of chronic diastolic dysfunction [11,12]. Increased LAV has been shown to be associated with cardiovascular risk burden and an increased risk of atrial fibrillation, stroke, heart failure and death [11-14]. Therefore, we hypothesized that an increased LAV and some echocardiographic indices of LV diastolic dysfunction would correlate with poor exercise capacity in patients with dyspnea of unknown origin.

## Methods

### Study population

Patients with the complaint of shortness of breath who were referred for treadmill exercise testing and transthoracic echocardiography at Siriraj Hospital were prospectively enrolled in the study. The exercise testing was performed immediately after the echocardiographic examination. Eligible patients were adults patients, in sinus rhythm, and able to exercise on the treadmill. The patients with known coronary artery disease, history of clinical heart failure, moderate or severe valvular heart disease, congenital heart disease, history of coronary revascularization or any prior cardiac surgery, LV systolic dysfunction (ejection fraction < 50%), significant pulmonary hypertension (right ventricular systolic pressure > 50 mmHg), end-stage renal disease waiting for renal transplantation, pulmonary, hepatic or hematologic diseases were excluded from the study. The positive result of treadmill exercise testing and the echocardiographic detection of regional wall motion abnormality were also the exclusion criteria. The institutional review board of Siriraj Hospital approved the study protocol. Informed consent was obtained from all patients.

### Echocardiography

Each participant underwent a comprehensive transthoracic echocardiographic examination with two-dimensional, M-mode, Doppler echocardiography and TDI. The measurement of echocardiographic parameters was performed on 3–5 consecutive cardiac cycles and the average was used for the analysis. The LV end-systolic and end-diastolic dimensions and wall thickness were measured using M-mode tracing. The LV end-systolic and end-diastolic volume and LV ejection fraction were determined using the Modified Simpson’s rule (biplane) [15]. The LV diastolic function was assessed by Doppler echocardiography of transmitral flow velocities and TDI of the mitral annulus. Peak early (E) and late (A) diastolic velocities of mitral inflow and deceleration time of E were measured using pulsed-wave Doppler with the sample volume at the tip of mitral valve. The TDI determination of diastolic function was performed in apical 4-chamber view with the sample volume at the septal aspect of mitral annulus. Longitudinal early (E’) and late (A’) diastolic myocardial velocities were measured. The E/A and E/E’ ratio were calculated. Left atrial (LA) dimension was measured by M-mode echocardiography from the parasternal short-axis view at end-systole. LAV was determined using the biplane area-length method and LV mass was calculated as recommended by the American Society of Echocardiography [15]. LAV and LV mass were indexed for body surface area. LA enlargement was defined as LAV index **≥** 29 ml/m^2^ as recommended by the American Society of Echocardiography [15]. Similarly, in the present study, the best cutoff value of LAV index for predicting good exercise capacity from the receiver operating characteristic curve (ROC) analysis was 29 ml/m^2^.

### Treadmill exercise testing

Symptom-limited treadmill exercise testing with the Bruce protocol was performed to assess the patients’ maximal exercise capacity. All patients were encouraged to exercise to maximal effort. The physician who was unaware of the echocardiographic results was present during exercise testing. Patients were instructed to continue their current medications on the day of testing. The predetermined end points of exercise testing were a positive test or maximal exercise capacity. Heart rate was recorded from a continuous 12-lead ECG monitoring. The age-predicted maximal heart rate was calculated as 220-age. Target heart rate was defined as 85% of the age-predicted maximal heart rate. The exercise time was recorded. Maximal exercise capacity was defined by the achieved metabolic equivalents (METs), which equal 3.5 mL of oxygen uptake per kilogram of body weight per minute and was estimated from exercise time as METs = 1.1 + (0.016 × exercise time in seconds) [16]. Patients with good exercise capacity were defined as those achieved ≥ 7 METs [16]. Rate pressure product was calculated as the product of heart rate and systolic blood pressure at peak exercise.

### Statistical analysis

Clinical, exercise and echocardiographic data were described using descriptive statistics, including means, standard deviation, number and percentage. The Student’s t-test was used to compare continuous variables between 2 groups of patients. Comparison of categorical variables was performed using the Chi-square test. The ROC analysis was performed to determine the cutoff values of LAV index for predicting good exercise capacity. Univariate binary logistic regression analysis was used to assess the relationship between good exercise capacity and other variables. Multiple binary logistic regression analysis was performed using metabolic equivalent (good exercise capacity) as the dependent variable. Risk factors or independent variables (age in years, body mass index, peak heart rate, LA diameter, LAV index, A, E’, E/E’, LV mass index) were selected on the basis of clinical and statistical significances. The model was fitted by backward stepwise method for variable selection in and out of the model. All p-values are reported as 2-tailed, except where otherwise indicated. A p-value of ≤ 0.05 was considered statistically significant. Statistical analyses were performed using SPSS statistical package version 18.0.

## Results

The study consisted of 111 patients (58.1 ± 9.2 years of age, 54.1% male). LA enlargement was observed in 59 (53.2%) patients. The patients were classified into 2 groups according to the presence of LA enlargement. Clinical characteristics in all patients and patients grouped according to the presence of LA enlargement are shown in Table 1. Patients with LA enlargement were significantly older. However, gender, body mass index, coronary risk factors and current medications were not significantly different between the 2 groups.

**Table 1:** Clinical characteristics of all patients and the comparisons between patients with and without left atrial enlargement

**Variables**	**Total (N = 111)**	**LAVI < 29 ml/m**^**2 **^**(N = 52)**	**LAVI ≥ 29 ml/m**^**2 **^**(N = 59)**	**P-value**
Age (years)	58.1 ± 9.2	55.9 ± 8.8	60.1 ± 9.2	0.018
Male gender	60 (54.1)	30 (57.7)	30 (50.8)	0.470
BMI (kg/m^2^)	25.7 ± 3.8	25.6 ± 3.3	25.7 ± 4.1	0.960
Hypertension	71 (64.0)	31 (59.6)	40 (67.8)	0.370
Diabetes mellitus	23 (20.7)	9 (17.3)	14 (23.7)	0.405
Smoking	9 (8.1)	5 (9.6)	4 (6.8)	0.732
Dyslipidemia	64 (57.7)	33 (63.5)	31 (52.5)	0.245
Betablocker	50 (45.0)	22 (42.3)	28 (47.5)	0.586
ACEI/ARB	40 (36.0)	19 (37.3)	21 (35.6)	0.857
Diuretics	9 (8.1)	3 (5.7)	6 (10.2)	0.503

Data are expressed as mean ± standard deviation and number (percentage).

P values are for comparisons between 2 groups.

ACEI/ARB = Angiotensin converting enzyme inhibitor/angiotensin receptor blocker; BMI = Body mass index; LAVI = Left atrial volume index.

### Echocardiographic data

All patients had good LV systolic function with LV ejection fraction of 71.0 ± 5.8%. The right ventricular systolic pressure was 31.7 ± 6.5 mmHg. All patients had diastolic dysfunction; abnormal relaxation and pseudonormalization pattern in 64.9% and 35.1%, respectively. No patient had an advanced stage of diastolic dysfunction (restrictive filling pattern). LAV index and LV mass index were 31.4 ± 10.5 ml/m^2^ and 110.2 ± 40.1 gm/m^2^, respectively. The echocardiographic data are present in Table 2. Patients with LA enlargement significantly presented with more LV hypertrophy, larger LV end-diastolic dimension and volume, and more severe diastolic dysfunction (lower E’ and higher E/E’) than those without.

**Table 2:** Echocardiographic parameters of all patients and the comparisons between patients with and without left atrial enlargement

**Variables**	**Total (n = 111)**	**LAVI < 29 ml/m**^**2 **^**(n = 52)**	**LAVI ≥ 29 ml/m**^**2 **^**(n = 59)**	**P-value**
LVDD (mm)	48.2 ± 4.8	47.1 ± 3.7	49.1 ± 5.4	0.026
LVSD (mm)	27.9 ± 5.3	27.7 ± 4.3	28.0 ± 6.0	0.814
IVSd (mm)	10.9 ± 2.6	10.3 ± 2.4	11.4 ± 2.7	0.025
LVEDV (ml)	68.8 ± 16.6	65.0 ± 13.9	72.1 ± 18.1	0.023
LVESV (ml)	20.2 ± 8.0	19.4 ± 7.5	20.8 ± 8.4	0.380
LV ejection fraction (%)	71.1 ± 5.8	70.7 ± 5.1	71.5 ± 6.4	0.450
LV mass index (g/m^2^)	110.2 ± 40.1	96.0 ± 22.1	123.0 ± 47.8	< 0.001
LA diameter (mm)	43.0 ± 6.1	39.6 ± 5.0	46.1 ± 5.3	< 0.001
E (cm/sec)	79.4 ± 23.7	76.1 ± 21.0	82.3 ± 25.8	0.168
A (cm/sec)	84.8 ± 23.5	80.5 ± 21.8	88.6 ± 24.4	0.068
E/A ratio	1.0 ± 0.4	1.0 ± 0.4	0.9 ± 0.3	0.549
DT (ms)	201.3 ± 40.7	197.7 ± 29.6	204.6 ± 48.6	0.364
S’ (cm/sec)	7.0 ± 1.3	7.2 ± 1.4	6.9 ± 1.3	0.136
E' (cm/sec)	6.1 ± 1.6	6.4 ± 1.7	5.8 ± 1.3	0.033
E/E’ratio	13.6 ± 4.6	12.1 ± 3.0	14.9 ± 5.4	0.001
Diastolic dysfunction				
- Abnormal relaxation	72 (64.9)	32 (61.5)	40 (67.8)	0.491
- Pseudonormalization	39 (35.1)	20 (38.5)	19 (32.2)	

Data are expressed as number (percentage) or mean ± standard deviation.

P values are for comparisons between 2 groups.

A = peak late diastolic velocity of mitral inflow; DT = deceleration time; E = peak early diastolic velocity of mitral inflow; E’ = tissue Doppler peak early diastolic velocity of medial mitral annulus; IVSd = interventricular septum wall thickness during diastole; LA = left atrium; LAVI = left atrial volume index; LV = left ventricle; LVDD = left ventricular end-diastolic diameter; LVSD = left ventricular end-systolic diameter; LVEDV = left ventricular end-diastolic volume; LVESV = left ventricular end-systolic volume; S’ = tissue Doppler peak systolic velocity of medial mitral annulus.

### Exercise hemodynamics

The results of exercise testing are presented in Table 3. Achieving target heart rate was reported in 57.7% of patients. More patients without LA enlargement achieved target heart rate than those with LA enlargement (69.2% vs. 47.5%, p = 0.021). Regarding exercise capacity, the number of patients who achieved target heart rate was similar between those with and without good exercise capacity (60.3% vs. 52.6%, p = 0.439). Good exercise capacity was reported in 65.8% of patients. More patients without LA enlargement had good exercise capacity than those with LA enlargement (82.7% vs. 50.8%, p < 0.001). The absolute exercise capacity was significantly better in patients without LA enlargement (8.3 ± 1.7 vs. 7.3 ± 2.0, p = 0.005). No serious complication occurred during treadmill exercise testing.

**Table 3:** Exercise parameters of all patients and the comparisons between patients with and without left atrial enlargement

**Variables**	**Total (n = 111)**	**LAVI < 29 ml/m**^**2 **^**(n = 52)**	**LAVI ≥ 29 ml/m**^**2 **^**(n = 59)**	**P-value**
Rest HR (beats/min)	70.7 ± 14.4	74.4 ± 15.7	67.5 ± 12.5	0.012
Peak HR (beats/min)	137.2 ± 24.5	145.1 ± 22.0	130.1 ± 24.5	0.001
Rest SBP (mmHg)	145.6 ± 19.3	139.9 ± 17.9	150.5 ± 19.4	0.003
Peak SBP (mmHg)	209.4 ± 40.9	204.7 ± 38.8	213.5 ± 42.6	0.258
Rest DBP (mmHg)	79.7 ± 12.8	78.1 ± 13.4	81.0 ± 12.3	0.236
Peak DBP (mmHg)	87.9 ± 18.7	86.8 ± 17.2	88.8 ± 20.1	0.583
Rate-pressure products	28566.9 ± 8300.4	29515.1 ± 7832.7	27731.2 ± 8671.9	0.282
Exercise duration (minutes)	6.8 ± 2.1	7.4 ± 1.8	6.3 ± 2.1	0.005
Exercise capacity (METs)	7.7 ± 1.9	8.3 ± 1.7	7.3 ± 2.0	0.005
Exercise capacity > 7 METs	73 (65.8)	43 (82.7)	30 (50.8)	< 0.001

Data are expressed as mean ± standard deviation and number (percentage).

P values are for comparisons between the 2 groups.

DBP = diastolic blood pressure; HR = heart rate; LAVI = left atrial volume index; METs = metabolic equivalents; SBP = systolic blood pressure.

### Determinants of exercise capacity

**Figure 1:**
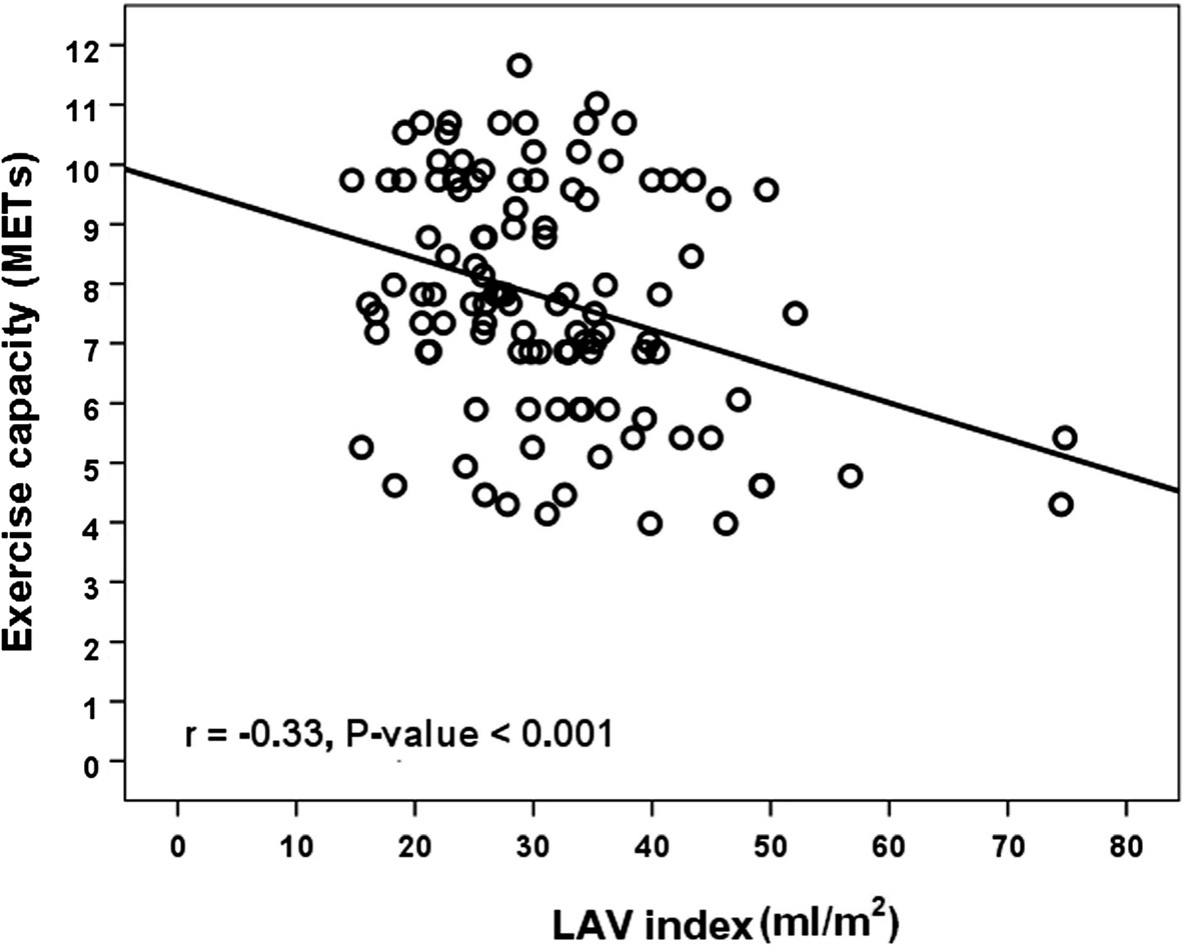
Correlation of left atrial volume (LAV) index with exercise capacity.

Regarding exercise capacity, the patients were further categorized as those with and without good exercise capacity. Table 4 shows the clinical, exercise and echocardiographic parameters of all patients and the comparisons between patients with and without good exercise capacity. There were no statistically significant differences between patients with and without good exercise capacity in term of the number of male gender, hypertension, diabetes mellitus, dyslipidemia, smoking, rest and peak systolic and diastolic blood pressure, and grade of diastolic dysfunction. The results of univariate and multivariate binary logistic regression analysis are shown in Table 5. LAV index remained an independent predictor of good exercise capacity. In univariate analysis, age, body mass index, peak heart rate, A, E’, E/E’ ratio, LA dimension, LV mass index and LAV index showed a significant association with good exercise capacity. By multiple logistic regression analysis, age [odds ratios (OR) 0.94; 95% confidence interval (CI) 0.89-0.99], body mass index (OR 0.82, 95% CI 0.72-0.93), and LAV index (OR 0.92, 95% CI 0.87-0.97) were associated with good exercise capacity (Table 5). In the present study, the cutoff value of LAV index of 29 ml/m^2^ predicted good exercise capacity with the sensitivity, specificity, predictive accuracy of positive and negative results of 76.3%, 58.9%, 49.2% and 82.7%, respectively, according to ROC analysis (AUC = 0.69 (95% CI: 0.59 to 0.80)). Furthermore, the patients were classified as either having or not having LA enlargement and categorized into 2 groups according to the cutoff value of LAV index of 29 ml/m^2^. Clinical characteristics, echocardiographic and exercise parameters in patients with and without LA enlargement are shown in Tables 1, 2 and 3, respectively. LAV index correlated with exercise capacity (r = −0.33, p < 0.001) (Figure 1).

**Table 4:** Clinical, exercise and echocardiographic parameters of all patients and the comparisons between patients with and without good exercise capacity

**Variables**	**Total (n = 111)**	**Exercise capacity < 7 METs (n = 38)**	**Exercise capacity ≥ 7 METs (n = 73)**	**P-value**
Age (years)	58.1 ± 9.2	61.7 ± 9.2	56.3 ± 8.7	0.003
Male gender	60 (54.1)	20 (52.6)	40 (54.8)	0.828
BMI (kg/m^2^)	25.7 ± 3.8	27.1 ± 4.0	24.9 ± 3.4	0.004
Rest HR (beats/min)	70.7 ± 14.4	70.8 ± 15.5	70.7 ± 14.0	0.975
Peak HR (beats/min)	137.2 ± 24.5	127.3 ± 23.2	142.3 ± 23.7	0.002
LVDD (mm)	48.2 ± 4.8	49.0 ± 5.5	47.8 ± 4.4	0.238
LVSD (mm)	27.9 ± 5.3	28.4 ± 6.3	27.6 ± 4.6	0.457
IVSd (mm)	10.9 ± 2.6	11.6 ± 3.1	10.5 ± 2.2	0.053
LVEDV (ml)	68.8 ± 16.6	71.7 ± 19.9	67.2 ± 14.4	0.175
LVESV (ml)	20.2 ± 8.0	21.4 ± 9.5	19.5 ± 7.0	0.220
LV ejection fraction (%)	71.1 ± 5.8	70.8 ± 6.4	71.3 ± 5.5	0.644
LV mass index (g/m^2^)	110.2 ± 40.1	124.4 ± 55.4	103.1 ± 27.2	0.032
LA diameter (mm)	43.0 ± 6.1	45.8 ± 5.2	41.7 ± 6.0	0.001
E (cm/sec)	79.4 ± 23.7	82.9 ± 24.1	77.6 ± 23.5	0.261
A (cm/sec)	84.8 ± 23.5	93.1 ± 24.7	80.5 ± 21.8	0.006
E/A ratio	1.0 ± 0.4	0.9 ± 0.3	1.0 ± 0.4	0.221
DT (ms)	201.3 ± 40.7	205.5 ± 48.8	199.1 ± 35.9	0.438
S’ (cm/sec)	7.0 ± 1.3	6.8 ± 1.3	7.2 ± 1.3	0.101
E' (cm/sec)	6.1 ± 1.6	5.6 ± 1.2	6.3 ± 1.7	0.007
E/E’ratio	13.6 ± 4.6	15.2 ± 5.5	12.7 ± 3.8	0.006
LAVI (ml/m^2^)	31.4 ± 10.5	36.3 ± 12.8	28.8 ± 8.0	0.002

Data are expressed as number (percentage) or mean ± standard deviation.

P values are for comparisons between 2 groups.

A = peak late diastolic velocity of mitral inflow; BMI = body mass index; DT = deceleration time; E = peak early diastolic velocity of mitral inflow; E’ = tissue Doppler peak early diastolic velocity of medial mitral annulus; HR = heart rate; IVSd = interventricular septum wall thickness during diastole; LA = left atrium; LAVI = left atrial volume index; LV = left ventricle; LVDD = left ventricular end-diastolic diameter; LVSD = left ventricular end-systolic diameter; LVEDV = left ventricular end-diastolic volume; LVESV = left ventricular end-systolic volume; METs = metabolic equivalents ; S’ = tissue Doppler peak systolic velocity of medial mitral annulus.

**Table 5:** Univariate and multivariate logistic regression analysis of parameters that associate with good exercise capacity

	**Univariate OR (95% CI)**	**P-value**	**Multivariate OR (95% CI)**	**P-value**
Age	0.93 (0.88 to 0.98)	0.004	0.94 (0.89 to 0.99)	0.018
Body mass index	0.85 (0.76 to 0.95)	0.005	0.82 (0.72 to 0.93)	0.002
Peak heart rate	1.03 (1.01 to 1.05)	0.003	-	
A	0.98 (0.96 to 0.99)	0.009	-	
E’	1.42 (1.06 to 1.91)	0.018	-	
E/E’ ratio	0.89 (0.81 to 0.97)	0.009	-	
LA dimension	0.88 (0.81 to 0.95)	0.001	-	
LV mass index	0.99 (0.98 to 0.99)	0.015	-	
LAVI	0.93 (0.88 to 0.97)	0.001	0.92 (0.87 to 0.97)	0.003

A = peak late diastolic velocity of mitral inflow; CI = confidence interval; E = peak early diastolic velocity of mitral inflow; E’ = tissue Doppler peak early diastolic velocity of medial mitral annulus; LA = left atrium; LAVI = left atrial volume index; LV = left ventricle; OR = odd ratio.

## Discussion

The present study emphasizes the importance of LAV index as an independent echocardiographic predictor of exercise capacity in patients with dyspnea of unknown origin. Among several echocardiographic variables of diastolic function, A, E’, E/E’ and LAV index correlated significantly with exercise capacity. Interestingly, LAV index was found to be the only echocardiographic measure of diastolic function that represents an independent predictor of exercise capacity in this population.

### Diastolic dysfunction and exercise capacity

Dyspnea on exertion or reduced exercise capacity is a major clinical problem in patients with LV diastolic dysfunction, more pronounced in diastolic heart failure. It is well known that LV diastolic dysfunction is common in patients with LV systolic dysfunction and Doppler variables of LV diastolic filling are better than LV systolic indices in predicting exercise capacity in patients with systolic heart failure [9,10]. Similarly, the correlations between the echocardiographic parameters of diastolic function and exercise capacity have been demonstrated in patients with isolated diastolic dysfunction [6,7,11,17-20]. Previous studies in patients with diabetes mellitus and hypertension showed that maximum exercise capacity was lower in those with diastolic dysfunction than in control subjects without diastolic dysfunction [17,18]. Several studies in patients with diastolic dysfunction, including the present study, demonstrated the strong and inverse association between exercise capacity/duration and some echocardiographic parameters of LV diastolic dysfunction, such as high E/E’ or LA enlargement [7,8,19,20]. Among several measurable variables of diastolic dysfunction used to predict exercise capacity, LAV has been demonstrated as the most reliable and robust since its size correlates closely with an impaired LV filling and reflects the chronicity and severity to diastolic dysfunction [11]. Arruda AL et al. reported that LAV index negatively correlated with exercise capacity and the correlation was strongest in patients with isolated diastolic dysfunction as compared to those with systolic dysfunction or control subjects [19]. The findings of the present study were consistent with those of previous studies and demonstrated that the larger LAV index was independently associated with exercise intolerance.

### Left atrial enlargement as a biomarker of poor exercise capacity

LA enlargement commonly occurs in the setting of LV diastolic dysfunction, is associated with the presence of cardiovascular disease and has been shown to be a poor prognostic indicator of adverse cardiovascular outcomes, such as atrial fibrillation, heart failure, stroke, and death [11-14]. In the absence of LV systolic dysfunction, pulmonary hypertension, significant valvular disease or pericardial disease, patients with LV diastolic dysfunction commonly present with dyspnea on exertion or limited exercise capacity, presumably due to elevated pulmonary capillary pressure and LV filling pressure [21]. It is well known that correlations exist between exercise capacity and Doppler-derived indices of LV diastolic dysfunction, such as Doppler transmitral flow velocities and TDI of the mitral annulus [5-8,18,19,21]. However, data regarding LAV in predicting exercise capacity are more limited. The echocardiographic measurement of LAV is simple and may better reflect the severity and cumulative effect of LV filling pressure over longer time rather than Doppler-derived LV diastolic indices alone. Pritchett AM et al. reported in a study of the general population that LAV index was closely associated with the severity of diastolic dysfunction assessed by Doppler echocardiography [14]. The mechanism of LA enlargement in patients with isolated diastolic dysfunction is less convincing, but it has been hypothesized that diastolic dysfunction occurs as a consequence of cardiovascular diseases, such as LV hypertrophy from hypertension, and leads to chronic diastolic atrial pressure overload. Ultimately, LA enlargement and symptom of dyspnea are evident in patients with isolated diastolic dysfunction. Also, LA enlargement becomes a reliable indicator of advanced diastolic dysfunction, regardless of LV systolic function, underlying cardiovascular disease or LV hypertrophy [14]. Previous studies have emphasized the prognostic significance of LA enlargement in different populations [11,13]. Furthermore, Tsang TS et al. demonstrated that LAV index had a positive correlation with LV filling pressure as indicated by E/E’ [11]. As exercise intolerance is a common clinical problem in patients with diastolic dysfunction, only few studies have demonstrated the association between LA enlargement and exercise capacity [19,22,23]. Previous study in patients with LV systolic dysfunction and heart failure showed that indexed LA diastolic and systolic size were independent predictors of exercise capacity and cardiovascular events (cardiac death or hospitalization for worsening heart failure), respectively [22]. However, the authors employed LA dimension, not LAV, in the study. LA remodeling is a continuous process as long as cardiovascular risk factors or diseases still persist in any individual. As chronic diastolic burden and LA remodeling continue over time, LA shape may become less spherical and LA volume may provide a more reliable and consistent representative of LA remodeling than LA dimension [12]. In a recent study by Vaturi M et al., LA enlargement, defined as LAV index > 42 ml/m^2^, was associated with poor exercise capacity [23]. However, the study was conducted in asymptomatic patients with chronic volume overload of severe mitral regurgitation, whereas LA enlargement was expected. Another study in patients with isolated diastolic dysfunction showed that elevated LAV was predictive of exercise capacity and elevated ventilator responses, similar to Doppler indices of diastolic function [19]. The present study demonstrated the similar finding that LAV index significantly correlated with exercise capacity in patients with normal systolic function and dyspnea on exertion of unknown origin. However, there was no correlation among the subgroup of patients without LA enlargement. This finding emphasized the importance of LAV index in risk stratification for patients with dyspnea and diastolic dysfunction, especially those with chronic diastolic dysfunction as manifested by LA enlargement. Furthermore, recent studies have focused on the measurement of LA function using the speckle tracking method in patients with diastolic dysfunction [24,25] and demonstrated that LA function was a significant correlate of exercise capacity, regardless of hemodynamic response to stress, in a large group of patients with preserved LV ejection fraction undergoing exercise echocardiography [25].

### Study limitations

The present study has some limitations; including the relatively small sample size and the lack of complete cardiopulmonary exercise testing. Although the measurement of maximal oxygen consumption with direct gas exchange may be more accurate in the assessment of exercise capacity, the calculation of achieved METs is widely accepted for determining function capacity in routine clinical practice and has been shown to predict long term mortality in a large group of patients [1]. There is the potential for a referral bias that the study population may be those who seek medical attention and have more comorbidities than general population. The present study was not conducted in patients undergoing exercise stress echocardiography, but instead compared rest echocardiographic parameters with exercise capacity in patients undergoing echocardiography and treadmill exercise test. The immediate post-exercise echocardiographic variables may be a better predictor of exercise capacity, but were not available in the present study. The study employed the positive result of treadmill exercise testing and the echocardiographic detection of regional wall motion abnormality, not the coronary angiogram as a gold standard, to exclude coronary artery disease. Finally, more recent data on LA function using speckle tracking have been published and revealed promising results [24,25]. The technique has not yet been introduced to day-to-day practice in the echocardiographic laboratory and the measurement of LA function was not performed in the present study. However, the role of LA function, in addition or combination with LAV, in predicting exercise capacity or clinical outcomes warrants further study.

## Conclusions

In patients with isolated diastolic dysfunction and exertional dyspnea, increased LAV index, a marker of chronic diastolic dysfunction, is associated with poor exercise capacity. Therefore, LAV index should be assessed as a part of routine echocardiographic examination and LA enlargement may become a part of clinical risk stratification in patients presented with exertional dyspnea.

## Abbreviations

CI: Confidence interval; LA: Left atrial; LAV: Left atrial volume; LV: Left ventricle; METs: Metabolic equivalents; OR: Odds ratios; ROC: Receiver operating characteristic; TDI: Tissue doppler imaging.

## Competing interests

The authors declare that they have no competing interests.

## Authors’ contributions

NR, conceived the study, participated in study design, analyzed data, interpreted exercise and echocardiographic data, drafted and revised the manuscript; KK, performed statistical analysis; SC, performed exercise testing and collected data; RK analyzed data, drafted and revised the manuscript; DJ, interpreted echocardiographic data. All authors read and approved the final manuscript.
